# A Rare Case of Sinonasal Ewing Sarcoma With Radiologic-Pathologic Correlation

**DOI:** 10.7759/cureus.43708

**Published:** 2023-08-18

**Authors:** Jennifer Worthy, Malika P Ganguli, Mahlon R Kile, Alexander M Satei, Nicholas D Mills

**Affiliations:** 1 Medicine, Ross University School of Medicine, Bridgetown, BRB; 2 Diagnostic Radiology, Trinity Health Oakland Hospital, Pontiac, USA; 3 Diagnostic Radiology, Wayne State University School of Medicine, Detroit, USA

**Keywords:** nasopharyngeal carcinoma, magnetic resonance imaging, computed tomography, sinonasal tumor pathology, ethmoid sinus, esthesioneuroblastoma, ewing sarcoma

## Abstract

An 89-year-old male presented with syncope and worsening difficulty in breathing through the left nostril. Computed tomography demonstrated a tumor in the anterior ethmoid air cells and maxillary sinus, which extended into the frontal lobe. Magnetic resonance imaging similarly demonstrated an aggressive lesion. This mass was difficult to differentiate from more commonly seen lesions at this location such as an esthesioneuroblastoma or nasopharyngeal carcinoma. Direct visualization, biopsy, and subsequent pathologic analysis eventually confirmed the diagnosis of malignant Ewing sarcoma (EWS).

Our case explores the radiological findings of EWS originating from the ethmoid sinus, compares EWS with other common carcinomas in the same location, confirms the diagnosis through pathological correlation, and investigates the prognosis and treatment of these lesions. This case highlights the importance of a multidisciplinary approach to diagnose EWS when it occurs in an atypical location. The clinical team relied on input from the radiology, surgery, ENT, neurology, and pathology departments to make an accurate diagnosis and plan treatment for this aggressive tumor.

## Introduction

Ewing sarcoma (EWS) is a rare malignant bone tumor that primarily affects the pelvis and lower extremities of children and adolescents. Rarely, EWS can develop in the sinonasal tract. The incidence of adult-onset EWS in the sinonasal tract is difficult to accurately assess due to the rarity of this cancer in adults and within this location. Head and neck cases of EWS range from 3-9% of all EWS cases [1]. A review of 183 cases of head and neck EWS demonstrated that the majority of these arose from the bones of the skull and face and subcutaneous soft tissues (128 cases or 70%); only one case (1%) arose from the nasopharynx [1]. One literature review in 2018 suggested only 10 cases of sinonasal or paranasal sinus EWS had been reported [2]. Typically, head and neck EWS demonstrate lower metastatic rates and tumor size compared to other EWS locations [1]. Given the exceeding low incidence of this disease in this location, cases of sinonasal EWS often require a multidisciplinary approach to diagnosis and treatment.

## Case presentation

History and examination

An 89-year-old man presented with syncope and progressive difficulty breathing through the left nostril. His past medical history was significant for coronary artery disease, benign prostate hyperplasia, and stage 3 chronic kidney disease.

A review of systems was negative for nosebleeds or headaches. Physical exam demonstrated symmetric facial movement, negative sinus tenderness, intact extraocular eye movement without nystagmus, normal cranial nerve II-XII function, and no lymphadenopathy. Nasal and oral examination showed no masses or polyps, with moist mucous membranes.

Imaging findings

**Figure 1:**
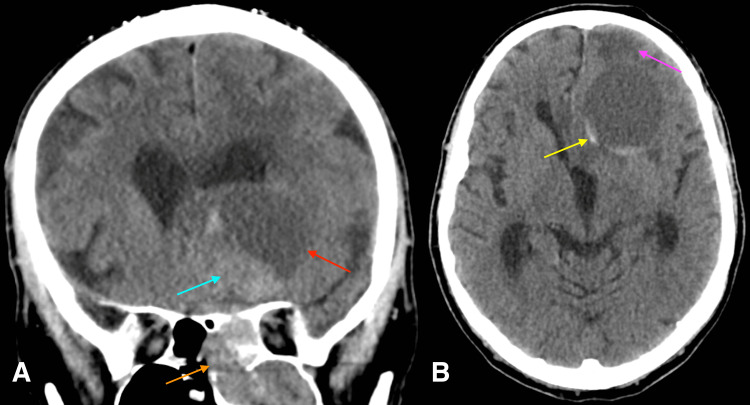
Computed tomography of the head without contrast in the coronal (A) and axial plane (B) Images demonstrate soft tissue opacification involving the left ethmoid and maxillary sinus (orange arrow), with extension into the anterior cranial fossa, where there is lobulated soft tissue (blue arrow) and a cystic component (red arrow); moderate vasogenic edema involving the frontal lobe anteriorly (pink arrow); and some peripheral hyperdensity associated with the cystic component of the lesion (yellow arrow), which could represent a small amount of blood product in the dependent aspect

The patient underwent computed tomography (CT) of the head without contrast (Figures 1a, 1b). Initial CT demonstrated a soft tissue mass within the anterior ethmoidal air cells and maxillary sinus with extension into the anterior cranial fossa. The intracranial portion of the mass contained pseudocystic components, with a curvilinear hyperdensity along the posterior medial wall consistent with internal hemorrhage or mineralization, and measured 5.6 x 4.8 x 5 cm. There was associated vasogenic edema along the anterior left frontal lobe. The localized mass effect resulted in near total effacement of the left lateral ventricle and left-to-right midline shift measuring approximately 1 cm.

**Figure 2:**
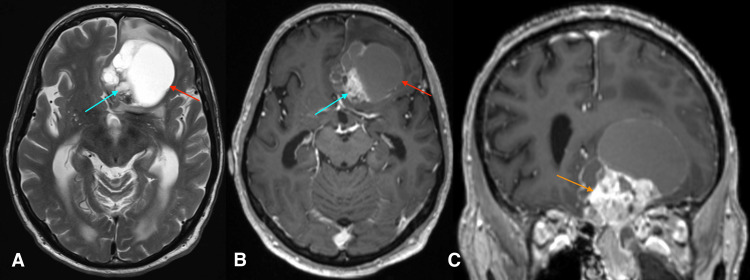
Magnetic resonance imaging of the brain T2-weighted in the axial plane (A), T1 post-gadolinium contrast in the axial (B) and coronal planes (C) T2-weighted imaging demonstrates a hyperintense cystic mass (red arrow) with a solid mural component within the medial left frontal lobe (blue arrow). Post-gadolinium imaging demonstrates a mixed cystic (red arrow) and solid mass with enhancement of the solid components along the posteromedial wall (blue arrow). The enhancing extra-axial soft tissue extends through the cribriform plate (orange arrow), severely distorting the adjacent frontal lobe.

The patient subsequently underwent magnetic resonance imaging (MRI) of the brain with contrast for further evaluation of the mass (Figures 2a-2c). There was a re-demonstration of a large mass originating from the left ethmoid air cells. This mass extended into the left nasal cavity and anterior cranial fossa and terminated predominantly along the medial left frontal lobe. The intracranial portion of the mass demonstrated diffusion restriction and was T1 and T2 hyperintense. Post-contrast imaging revealed enhancement of the extra-axial soft tissue components of the mass.

Histological analysis

The patient was referred to the ear, nose, and throat service. Flexible fiberoptic laryngoscopy was performed, demonstrating a mass superior and medial to the middle turbinate with normal surrounding mucosa and no ulceration, correlating with the mass visualized on cross-sectional imaging. Biopsy samples were taken and sent for histological review.

**Figure 3:**
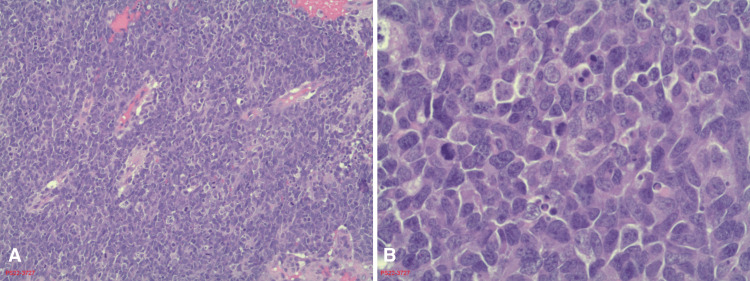
Low-power (A) and high-power (B) H&E stain Low-power H&E demonstrates a sheet-like pattern of small round blue cells. Mitotic figures and apoptotic bodies can be seen. High-power H&E demonstrates pleomorphic cells with large, round to oval nuclei with fine chromatin and a few with prominent nucleoli. There is a high nuclear-to-cytoplasmic ratio with pink cytoplasm. H&E: hematoxylin and eosin

**Figure 4:**
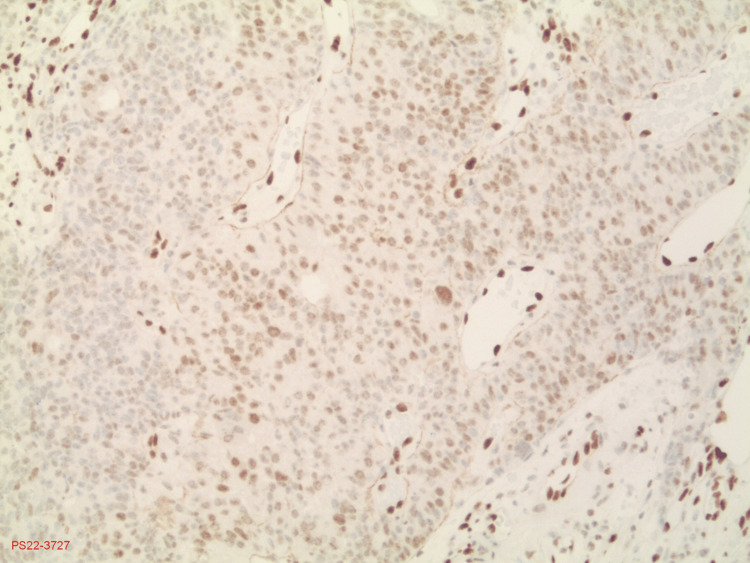
FLI-1 stain This stain shows nuclear activity. The darker staining endothelial cells are a normal finding while the tumor cells have taken up a lighter nuclear stain. FLI-1: friend leukemia integration 1 transcription factor

**Figure 5:**
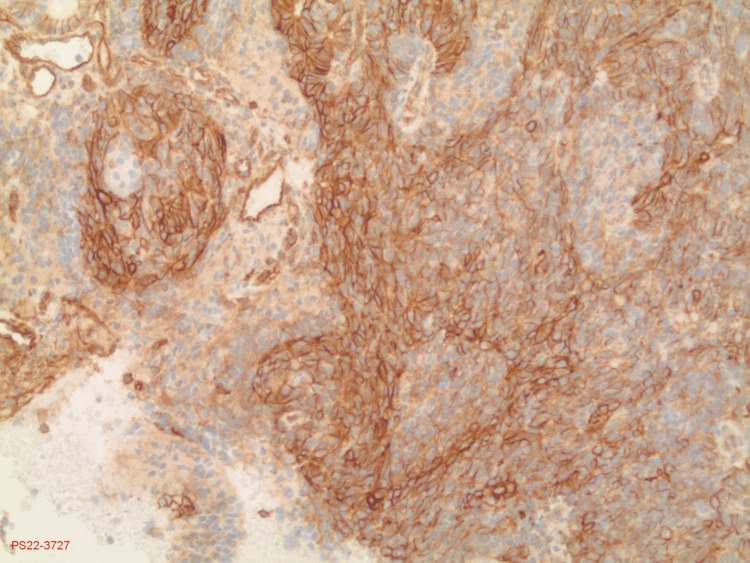
CD99 stain The stain shows a diffusely positive membrane. CD99 is highly sensitive for EWS; however, it is not specific. CD99: cluster of differentiation 99

Differential diagnoses for the mass included esthesioneuroblastoma (ENB), nasopharyngeal carcinoma, angiofibroma, and squamous cell carcinoma. Left nasal biopsy showed undifferentiated small, round, blue cells with apoptotic bodies and mitotic figures (Figures 3a, 3b). Immunohistochemical staining (IHC) was positive for vimentin, NSE, S-100, FLI-1 (Figure 4), cyclin D1, CD56, CD99 (Figure 5), and KI67 >95%. In addition, IHC showed patchy positivity for synaptophysin and p16. The biopsy provided a definitive diagnosis of malignant Ewing sarcoma. The patient decided not to pursue treatment and passed away shortly after discharge.

## Discussion

Ewing sarcoma (EWS) is a primary bone and soft tissue tumor typically occurring in children and adolescents and usually originating within the medullary cavity of long or flat bones [3]. Common locations include the pelvis and lower extremities. EWS may also arise in several different visceral locations [4]. This tumor is highly malignant and rapidly invades the cortex. Additionally, the tumor typically affects males more than females in a 3:2 ratio and is less commonly seen in Black or Asian populations. It is the second most common bone tumor in childhood [5]. Initially, patients generally experience localized pain and swelling. Fever, leukocytosis, elevated erythrocyte sedimentation rate, and elevated lactate dehydrogenase may also be present.

In 85% of EWS cases, a t(11;22) chromosomal translocation is found. The EWSR1 gene from chromosome 22 fuses with the FLI-1 gene on chromosome 11 [6]. This fusion drives cellular proliferation through activated transcription factors, causing overwhelming growth. EWS is therefore extremely aggressive with early metastasis to other bones and lung tissue.

A characteristic feature of Ewing sarcoma is the presence of a periosteal reaction, typically referred to as an “onion skin-like” or lamellated pattern of new bone formation. On CT imaging, Ewing sarcoma typically appears as an aggressive heterogeneous mass with poorly defined lucent lesions and margins sparing the cortex. On MRI, there is intermediate to high signal intensity on T2-weighted images and low to intermediate signal intensity on T1-weighted images [7]. In contrast, the main differential of this case considering the location, ENB typically appears as a soft tissue mass within the sinonasal cavity with erosion of the adjacent bones and extension through the cribriform plate. On CT, ENB typically shows homogeneous enhancement and focal calcifications. There is less aggressive destruction of the bony margins when compared to Ewing sarcoma. On MRI, ENB demonstrates heterogeneous intermediate signals on T1 and T2-weighted imaging [8].

The histological differences between EWS and the differential diagnoses in this case ultimately demonstrated the importance of multidisciplinary radiologic-pathologic teamwork. EWS typically demonstrates uniform, small, round cells with round nuclei and stippled chromatin. There is a high nuclear-to-cytoplasm ratio and mitotic figures are sparsely present. Additionally, the tumor cells contain abundant glycogen within the cytoplasm with minimal intervening stroma between cells. In the majority of cases, EWS stains positive for CD99, vimentin, FLI-1, and NKX2.2, the combination of which carries high specificity for the disease [9]. Neuroendocrine markers may be variable in EWS. For instance, it is not uncommon for EWS to stain positive for synaptophysin, CD56, and neuron-specific enolase (NSE). Generally, EWS does not stain positively with a chromogranin stain [10]. In contrast to Ewing sarcoma, ENB will stain positively for synaptophysin, chromogranin, CD56, neurofilament, and S100 [11]. Additionally, ENB will stain negatively for CD99 and FLI-1, which were positive in this case. Finally, ENB on histological analysis will demonstrate lobules of tumor cells with small round nuclei. Nasopharyngeal carcinoma would also show a different pattern of IHC staining: positive cytokeratin, positive p63, positive p16, positive EMA, and negative NSE [12]. Table 1 discusses how the selected IHC markers support EWS as the diagnosis in this case and makes all other differential diagnoses for this tumor less likely [11-14].

**Table 1:** Our patient’s tumor findings A. The tumor showed positivity for neuroendocrine stains, except for chromogranin (CHR). Positive CHR would be unusual in EWS [13]. B. S100 was positive, a sensitive, nonspecific marker for melanoma. HMB45 and MART-1 are specific for melanoma, which were negative. C. Vimentin is known to be nonspecific but may be helpful in narrowing into sarcoma, lymphoma, or melanoma categories [11]. Most supportive of EWS is positivity for CD99 and FLI-1, which make nasopharyngeal carcinoma [12] and olfactory neuroblastoma [14] less likely. D. While p16 is positive, a surrogate marker for HPV, this tumor is completely negative for cytokeratin. E. Lymphomas and leukemias may show positive CD99. However, specific markers for B- and T-cells were negative. EWS: Ewing sarcoma, NE: neuroendocrine; HPV: human papillomavirus; SqCC: squamous cell carcinoma

	Tumor Immunohistochemistry Results	
	Positive	Negative
A.	SYN (patchy), CD56, NSE	CHR
B.	S100	HMB45, MART-1
C.	Vimentin, CD99, FLI-1	-
D.	p16 (patchy)	AE1/AE3, EMA, p63, CK5/6
E.	-	CD3, CD10, CD20, CD10

Treatment for Ewing sarcoma also involves a multidisciplinary approach. Neoadjuvant chemotherapy of alternating vincristine, doxorubicin, cyclophosphamide (VDC), and ifosfamide and etoposide (IE) is considered the standard of care in the United States. Slightly different chemotherapy regimens have also proven successful, such as the exclusion of cyclophosphamide or just relying on VDC without the use of alternating IE, depending on the shared decision-making between the oncologist and patient. These chemotherapy agents are typically used in combination with targeted radiotherapy of the primary site. Patients who received radiation therapy for metastatic EWS have better three-year event-free survival than those who do not (35% versus 16%) [15]. Excision can be considered in localized disease, however, the extent and area involved in this case would make it virtually impossible. Even with treatment, the prognosis remains poor in cases of sinonasal EWS.

## Conclusions

Sinonasal Ewing sarcoma represents an exceedingly rare diagnosis, particularly in older populations. This, in conjunction with the various other differentials most commonly seen within the sinonasal tract, makes diagnosis on cross-sectional imaging difficult. This case highlights the importance of maintaining an index of suspicion for EWS when considering the differential diagnosis of a highly aggressive appearing sinonasal mass. Additionally, this case demonstrates the radiologic-pathologic correlation required for a definitive diagnosis and for appropriate surgical and oncological treatment planning.
